# The combination of ANT2 shRNA and hNIS radioiodine gene therapy increases CTL cytotoxic activity through the phenotypic modulation of cancer cells: combination treatment with ANT2 shRNA and I-131

**DOI:** 10.1186/1471-2407-13-143

**Published:** 2013-03-22

**Authors:** Yun Choi, Ho Won Lee, Jaetae Lee, Yong Hyun Jeon

**Affiliations:** 1 Department of Pathology, Seoul National University College of Medicine, Seoul, Korea; 2 Department of Nuclear Medicine, Kyungpook National University, 807 Hogukro, Bukgu, Daegu, 700-721, Republic of Korea; 3 Leading-edge Research Center for Drug Discovery and Development for Diabetes and Metabolic Disease, Kyungpook National University, 807 Hogukro, Bukgu, Daegu, 700-721, Republic of Korea

**Keywords:** Human sodium iodide symporter (hNIS), Radioiodine gene therapy, Adenine nucleotide translocase-2 (ANT2), Short hairpin RNA (shRNA), Radiation-induced immune response, Cytotoxic T cells (CTLs)

## Abstract

**Background:**

It is important to simultaneously induce strong cell death and antitumor immunity in cancer patients for successful cancer treatment. Here, we investigated the cytotoxic and phenotypic modulation effects of the combination of ANT2 shRNA and human sodium iodide symporter (hNIS) radioiodine gene therapy in vitro and in vivo and visualized the antitumor effects in an immunocompromised mouse colon cancer model.

**Methods:**

A mouse colon cancer cell line co-expressing hNIS and the luciferase gene (CT26/hNIS-Fluc, named CT26/NF) was established. CT26/NF cells and tumor-bearing mice were treated with HBSS, scramble, ANT2 shRNA, I-131, and ANT2 shRNA + I-131. The apoptotic rates (%) and MHC class I and Fas gene expression levels were determined in treated CT26/NF cells using flow cytometry. Concurrently, the level of caspase-3 activation was determined in treated cells in vitro. For in vivo therapy, tumor-bearing mice were treated with scramble, ANT2 shRNA, I-131, and the combination therapy, and the anti-tumor effects were monitored using bioluminescence. The killing activity of cytotoxic T cells (CTLs) was measured with a lactate dehydrogenase (LDH) assay.

**Results:**

For the in vitro experiments, the combination of ANT2 shRNA and I-131 resulted in a higher apoptotic cell death rate compared with ANT2 shRNA or I-131 alone, and the levels of MHC class I and Fas-expressing cancer cells were highest in the cells receiving combination treatment, while single treatment modestly increased the level of MHC class I and Fas gene expression. The combination of ANT2 shRNA and I-131 resulted in a higher caspase-3 activation than single treatments. Interestingly, in vivo combination treatment led to increased gene expression of MHC class I and Fas than the respective mono-therapies; furthermore, bioluminescence showed increased antitumor effects after combination treatment than monotherapies. The LDH assay revealed that the CTL killing activity against CT26/NF cells was most effective after combination therapy.

**Conclusions:**

Increased cell death and phenotypic modulation of cancer cells in vitro and in vivo were achieved simultaneously after combination therapy with ANT2 shRNA and I-131, and this combination therapy induced remarkable antitumor outcomes through improvements in CTL immunity against CT26/NF. Our results suggest that combination therapy can be used as a new therapeutic strategy for cancer patients who show resistance to single therapy such as radiation or immunotherapy.

## Background

To achieve successful cancer treatment, it is important to overcome obstacles that occur when cancer patients receive single treatment such as chemotherapy, radiation therapy and immunotherapy. For example, resistance to chemotherapeutic drugs or radiation can cause cancer treatment failure. In addition, during immunotherapy, several impediments (such as tumor-derived cytokine suppression, loss of danger signals and MHC class molecules, and reduced antigen expression) in the micro-tumor environment allow cancer cells to escape from immune surveillance [1-5]. Due to the limitations of single therapy, new combined therapies that can simultaneously induce strong cytotoxic effects and enhance anti-tumor immunity should be explored.

Among the different subtypes of ANT (ANT1-4), ANT2 is over-expressed in proliferative cells, and the induction of ANT2 is directly involved in the glycolytic metabolism of cancer cells [6,7]. Previously, we demonstrated antitumor effects in melanoma mouse cancer cells and a xenograft model through ANT2 inhibition using siRNA technology [8]. Interestingly, ANT2 inhibition with RNAi induces a phenotypic modulation of cancer cells such as alterations in Fas, MHC class I, and ICAM-I expression levels, and sequentially, these modulations enhance anti-tumor immunity when combined with hMUC1 DNA vaccination.

The sodium/iodide symporter gene (NIS) is a specialized active iodide transporter [9,10]. Transfection of the NIS gene into tumor xenografts facilitates the accumulation of therapeutic (I-131 and Re-188) or diagnostic (I-123 and Tc-99 m) radioisotopes for the simultaneous imaging and treatment of cancer [11-13]. Similar to ANT2 shRNA treatment, we showed that hNIS radioiodine gene therapy modulates the phenotype of cancer cells in vitro and in vivo, resulting in an increased susceptibility of cancer cells to cytotoxic T cells (CTLs) [14].

Based on our reports, we considered that because both hNIS radioiodine gene therapy and ANT2 RNAi have therapeutic advantages in not only inducing strong apoptosis but also in simultaneously increasing anti-tumor immunity, further studies were required to determine the potential therapeutic effects of their combination treatment in vitro and in vivo.

Herein, we attempted to investigate the following: 1) whether the combination of ANT2 shRNA and hNIS radioiodine gene therapy can induce more effective cytotoxic effects and phenotypic modulation in a mouse colon cancer model in vitro and in vivo; and 2) whether combination therapy can enhance the antitumor immunity of CTLs and tumor growth inhibition effects.

## Methods

### Animals

Pathogen-free six-week-old female Balb/c mice were obtained from SLC Inc. (Japan). All animal experiment protocols were approved by the Committee for the Handling and Use of Animals, Kyungpook National University.

### Cell lines and DNA constructs

CT26, an adenocarcinoma colon cancer cell line that co-expresses the hNIS and firefly luciferase genes was established using a retro and lentiviral system (referred to as CT26/NF cells), and gene expression in this cell line was confirmed through ^125^I uptake and luciferase assays (data not shown).

The scramble or ANT2 shRNA DNA vector has been previously described in detail [8]. Plasmid DNA was amplified in Escherichia coli DH5α cells and purified through large-scale plasmid preparation using endotoxin-free Giga Prep columns (Qiagen, Chatsworth, CA). The DNA was dissolved in endotoxin-free buffer for storage.

### Apoptosis analysis

For apoptosis analysis after I-131, the CT26/NF cells were grown in 75-cm^2^ flasks and incubated for 7 h at 37°C in HBSS only or HBSS containing 0.05, 0.3, and 0.6 mCi/5 mL Na^131^I. The reaction was terminated by removing the radioisotope-containing medium and washing the cells twice with HBSS. For apoptosis analysis after ANT2 shRNA, the CT26/NF cells were grown in a six-well plate and transfected with scramble or ANT2 shRNA (0.1, 1, and 10 ug) using Lipofectamine 2000 (Invitrogen, Carlsbad, CA, USA). For apoptosis analysis after a combination of I-131 and ANT2 shRNA, the CT26/NF cells were grown in 75-cm^2^ flasks and incubated for 7 h at 37°C in HBSS only or HBSS containing 0.3 mCi/2 mL Na^131^I and then transfected with scramble or ANT2 shRNA (0.1, 1, and 10ug) using Lipofectamine 2000 (Invitrogen, Carlsbad, CA, USA). Two days after transfection, the cells were harvested and stained with a solution of FITC-conjugated Annexin V and propidium iodide (BD Pharmingen, San Diego, CA, USA). Flow cytometric analysis was performed using a Becton-Dickinson FACScan and CELLQuest software (Becton Dickinson Immunocytometry Systems, CA).

### Measurement of caspase activity

Caspase 3/7 activities were measured by using the Caspase-Glo 3/7 assay Kit (Promega, Madison, WI) following manufactures instructions. Briefly, the proluminescent substrate containing the DEVD (the sequence is in a single-letter amino acid code) is cleaved by caspase-3/7. After caspase cleavage, a substrate for luciferase (aminoluciferin) is released. This results in the luciferase reaction and the production of luminescent signal. CT26/NF cells were grown in 75-cm^2^ flasks and incubated for 7 h at 37°C in HBSS only or HBSS containing 0.3 mCi/2 mL Na^131^I and then transfected with scramble or ANT2 shRNA (0.1, 1, and 10ug) using Lipofectamine 2000 (Invitrogen, Carlsbad, CA, USA). At 2 days after treatments, cells were lysed and substrate cleavage by caspases was measured by the generated luminescent signal with a 96 multi-well luminometer (Molecular Devices, Sunnyvale, CA). Each experiment was performed in quintuplicate and experiments were carried out twice.

### Phenotypic marker analysis

For the in vitro analysis of MHC class I and Fas receptor gene expression levels in cancer cells, 2×10^5^ cells were grown in 75-cm^2^ flasks and incubated for 7 h at 37°C in HBSS or HBSS containing 0.3 mCi/2 mL Na^131^I and then transfected with scramble or ANT2 shRNA (0.1, 1, and 10 ug) using Lipofectamine 2000 (Invitrogen, Carlsbad, CA, USA). Two days later, the treated cells were stained with PE-conjugated monoclonal rat anti-mouse MHC class I (BD Pharmingen, NJ) or PE-conjugated monoclonal hamster anti-mouse Fas.

To determine MHC class I and Fas receptor gene expression levels in the CT26/NF mouse tumor model, CT26/NF cells were subcutaneously implanted into the right thighs of mice (7 mice/group). Scramble and ANT2 shRNA (100 ug/100 ul PBS) were intratumorally injected into tumor-bearing mice once per day for 3 days; then, the mice received I-131 (0.5 mCi) intravenously. Two days after I-131 administration, the mice were sacrificed, and the tumor masses were extracted. The tumors were dissociated using collagenase D (Roche), and single cells were stained with PE-conjugated monoclonal rat anti-mouse MHC class I (BD Pharmingen, NJ) or PE-conjugated monoclonal hamster anti-mouse Fas. Flow cytometric analysis was performed using a Becton-Dickinson FACScan unit using CELLQuest software (Becton Dickinson Immunocytometry Systems, CA).

### In vivo combination therapy and bioluminescence

CT26/NF cells were subcutaneously implanted into the right thighs of mice (7 mice/group). Scramble and ANT2 shRNA (100 ug/100 ul PBS) were intratumorally injected into tumor-bearing mice once per day for 3 days; then, the mice received I-131 (0.5 mCi) intravenously. To visualize the antitumor effect, bioluminescence was performed at a designated time point. An IVIS Lumina II (Caliper Life Sciences, MA, USA) was used for BLI acquisition and analysis. D-luciferin potassium salt (Caliper Life Sciences, MA, USA) was diluted to 3 mg/100 μl in PBS before use, and the mice were injected intraperitoneally with 100 μl of the D-luciferin solution. The BLI was obtained from the mice and analyzed using Living Image® (WaveMetrics, OR, USA). To quantify the emitted light, ROIs were drawn over the tumor region. The tumor sizes were measured using a caliper at 14, 21, 28 and 35 days post-inoculation. Tumor volumes were calculated using the formula *V* = 1/2 (*L* × *W*^2^), where L is the length (longest dimension), and W is the width (shortest dimension). On day 35, the tumors were excised and weighed.

### Cytotoxicity assays

The CytoTox 96 non-radioactive cytotoxicity assay (Promega, Madison, WI) was used to measure the cytotoxic activity levels of splenocytes in treated mice (7 mice/group) according to the manufacturer’s protocol with minor modifications. Briefly, the splenocytes of treated immunocompetent BALB/c mice were incubated in the presence of human IL-2 (50 U/ml) and irradiated CT26/NF and B16F10 cells (5 × 10^6^). After 3 days, the irradiated CT26/NF and B16F10 target cells were plated at 1×10^4^ cells/well on 96-well U-bottomed plates (Costar), and the splenocytes (effectors) were added to a final volume of 100 μl in ratios of 1:5, 1:15, and 1:30 (target to effector). The plates were then incubated for 4 hr in a humidified 5% CO_2_ chamber at 37°C and centrifuged at 500 *g* for 5 min. Aliquots (50 μl) were transferred from all wells to fresh 96-well flat-bottom plates, and an equal volume of reconstituted substrate mix was added per well. The plates were then incubated in the dark at room temperature for 30 min. Stop solution (50 μl) was added, and the absorbance was measured at 492 nm. The cell death percentages at each effector-to-target cell ratio were calculated using the following formula: [*A*_492*nm*_(*experimental*) − *A*_492*nm*_(*effector spontaneous*) − *A*_492*nm*_(*target spontaneous*)] × 100/[*A*_492*nm*_(*target maximum*) − *A*_492*nm*_(*target spontaneous*)].

### Statistical analyses

All data are expressed as the mean ± SD and are representative of at least triplicate experiments. The significance was determined using an unpaired Student’s *t* test. A value of *p* < 0.05 was considered to be significant.

## Results

### Combination with ANT2 shRNA and hNIS radioiodine gene therapy induced higher apoptosis levels than single treatment in vitro

**Figure 1:**
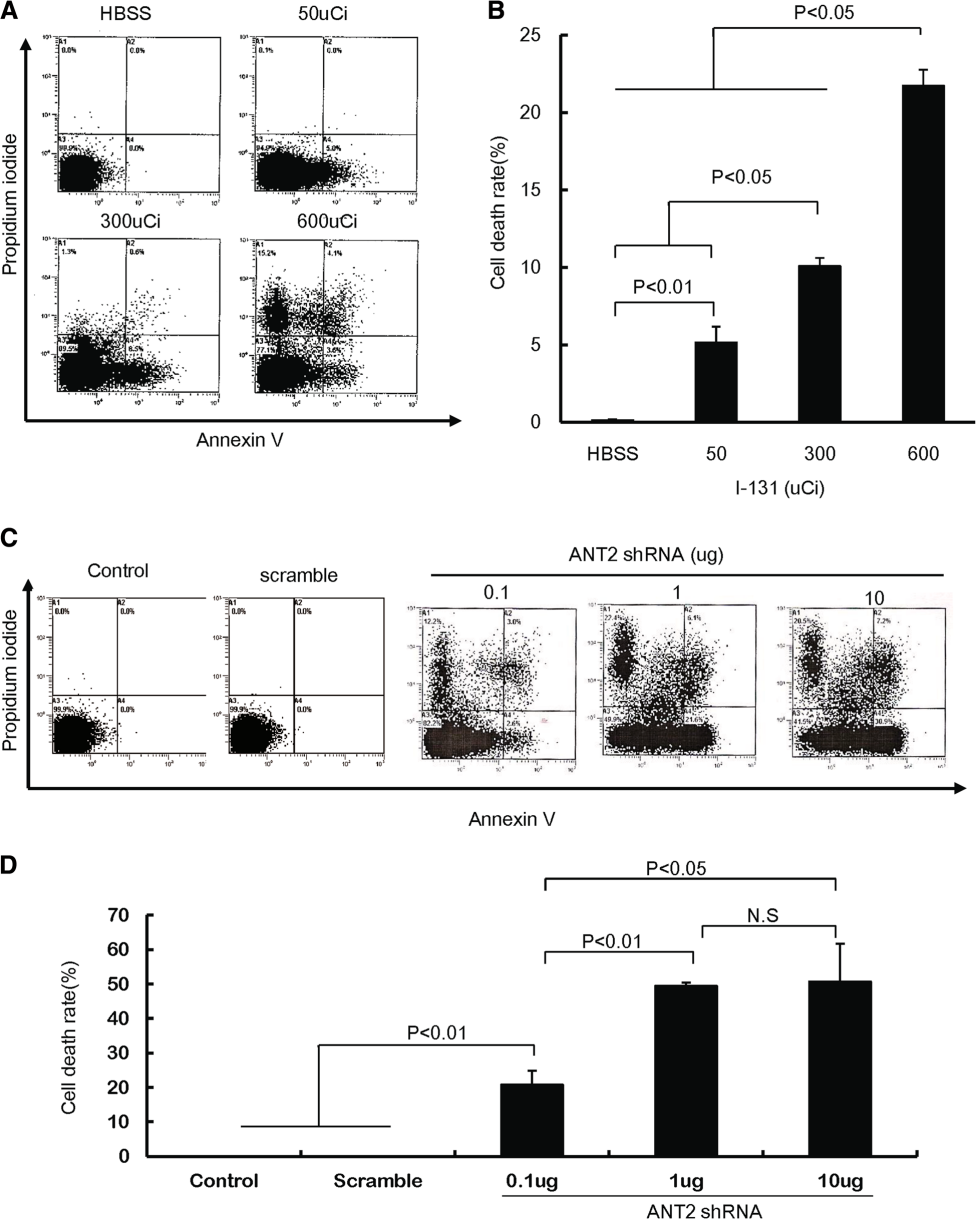
**The cytotoxic effects of ANT shRNA or hNIS radioiodine gene therapy in CT26/NF cells. (A)** and **(C)** Representative flow cytometry data for propidium iodide and Annexin V staining are shown. **(B)** and **(D)** The Y axis indicates the relative cell death (%), which is the sum of the early apoptotic portion (AV+PI-), the intermediate apoptotic portion (AV+PI+), and the late apoptotic portion (AV-PI-). The treated cells were stained with propidium iodide and FITC-conjugated Annexin V and analyzed using flow cytometry. The data shown are the mean of triplicate experiments; the bars represent the mean ± SD.

To determine whether I-131 treatment induced cell death through apoptosis, CT26/NF cells were treated with I-131 in HBSS at doses of 50, 300, and 600 μCi, and cell death was analyzed using FACS analysis. As shown in Figures 1A and B, the cell death rate (%) was increased in I-131-treated cells in a dose-dependent manner compared with HBSS-treated cells.

We next tested whether treatment with ANT2 shRNA induced cell death in CT26/NF cells in vitro. CT26/NF cells were transfected with scramble and ANT2 shRNA vector at different doses, and Annexin V- and propidium iodide-positive cells were determined using FACS analysis. Transfection of scramble shRNA failed to induce cell death in CT26/NF cells. However, robust cell death was detected in CT26/NF cells transfected with ANT2 shRNA vector in a DNA dose-dependent manner (Figures 1C and D; scramble shRNA and 0.1, 1, and 10 μg of ANT2 shRNA induced cell death rates of 0±0%, 20.7±4.1%, 49.4±0.9%, and 50.7±11.0%, respectively; P<0.01, control or scramble versus 0.1 μg ANT2 shRNA; P<0.01, 0.1 μg ANT2 shRNA versus 1 μg ANT2 shRNA).

**Figure 2:**
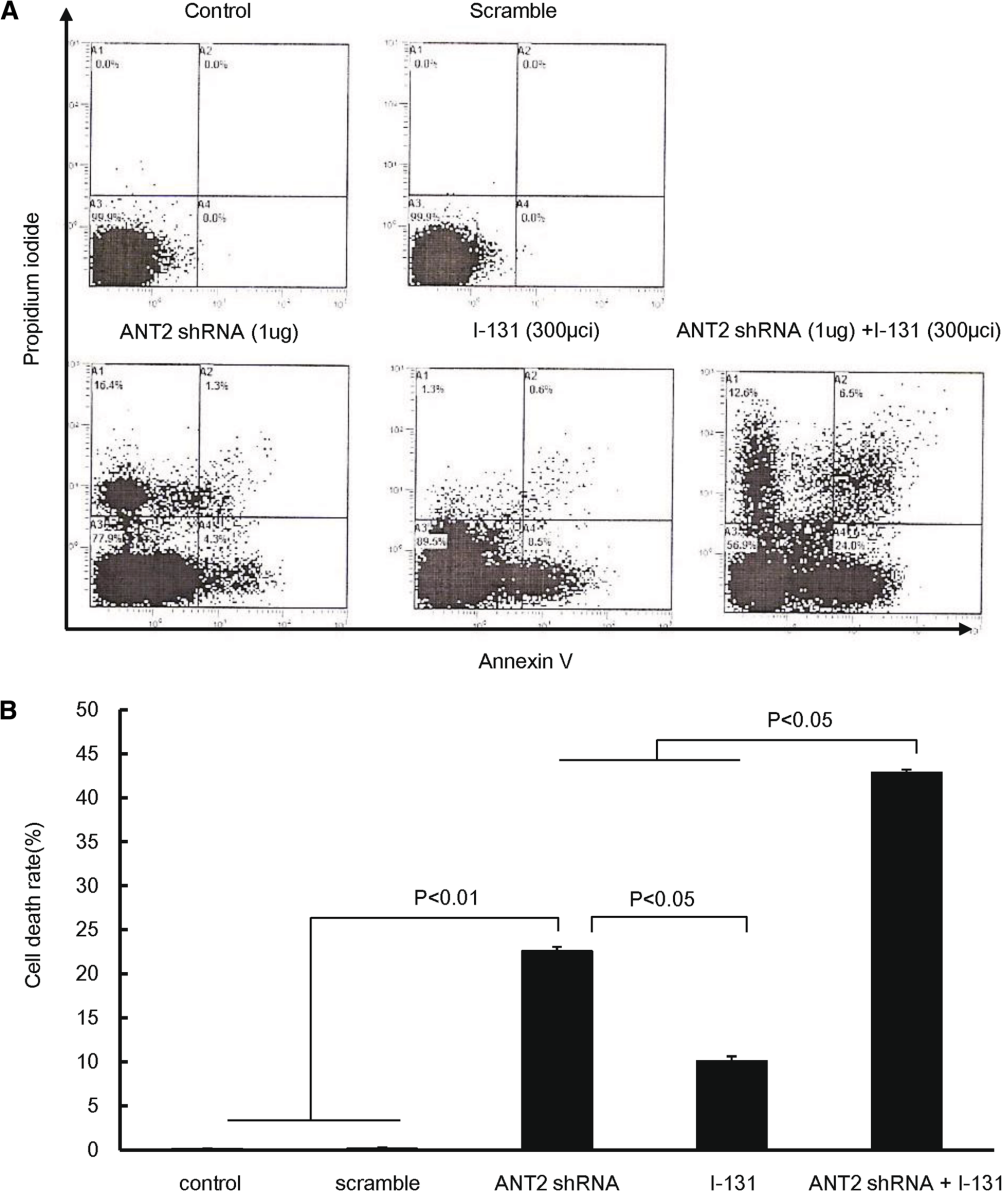
**Enhanced cytotoxicity with ANT shRNA and hNIS radioiodine combination therapy in CT26/NF cells. (A)** Representative flow cytometry data for propidium and Annexin V staining are shown. **(B)** The Y axis indicates the relative cell death (%), which is the sum the early apoptotic portion (AV+PI-), the intermediate apoptotic portion (AV+PI+), and the late apoptotic portion (AV-PI-). The treated cells were stained with propidium iodide and FITC-conjugated Annexin V and analyzed using flow cytometry. The data shown are the mean of triplicate experiments; the bars represent the mean ± SD.

We then investigated whether combination treatment showed enhanced cytotoxic effects in CT26/NF cells in vitro. CT26/NF cells were incubated with either HBSS or 300 μCi I-131 for 7 h and then transfected with either scramble shRNA or ANT2 shRNA. As shown in Figure 2, treatment with ANT shRNA or I-131 alone resulted in 22.6±0.5% and 10.1±0.5% cell death, respectively (P<0.01, control or scramble versus ANT2 shRNA; P<0.05, ANT2 shRNA versus I-131). The combined treatment resulted in a higher cell death rate (42.9±0.3%) than either single treatment (P<0.05, ANT2 shRNA or I-131 versus ANT2 shRNA+I-131). To further investigate whether the PI and Annexin V-positive cells detected with FACS analysis were truly apoptotic, we examined caspase-3 activation in treated cells because caspase-3 is the key factors in apoptosis. As shown in Additional file 1: Figure S1, treatment with ANT2 shRNA or I-131 alone leaded to more increased caspase-3 activation compared to control, respectively (P<0.05, control/or scramble versus ANT2 shRNA/ or I-131). Subsequently, the combination of ANT2 shRNA and I-131 resulted in a higher caspase-3 activation than single treatment (P<0.05, ANT2 shRNA or I-131 versus ANT2 shRNA+I-131).

### Combination therapy is more effective for the phenotypic modulation of cancer cells than single treatment in vitro and in vivo

To evaluate whether combination treatment induced a phenotype modulation of cancer cells, CT26/NF cells were treated with the same treatment procedure as in Figure 2, and the MHC class I and Fas expression levels were determined using FACS analysis.

**Figure 3:**
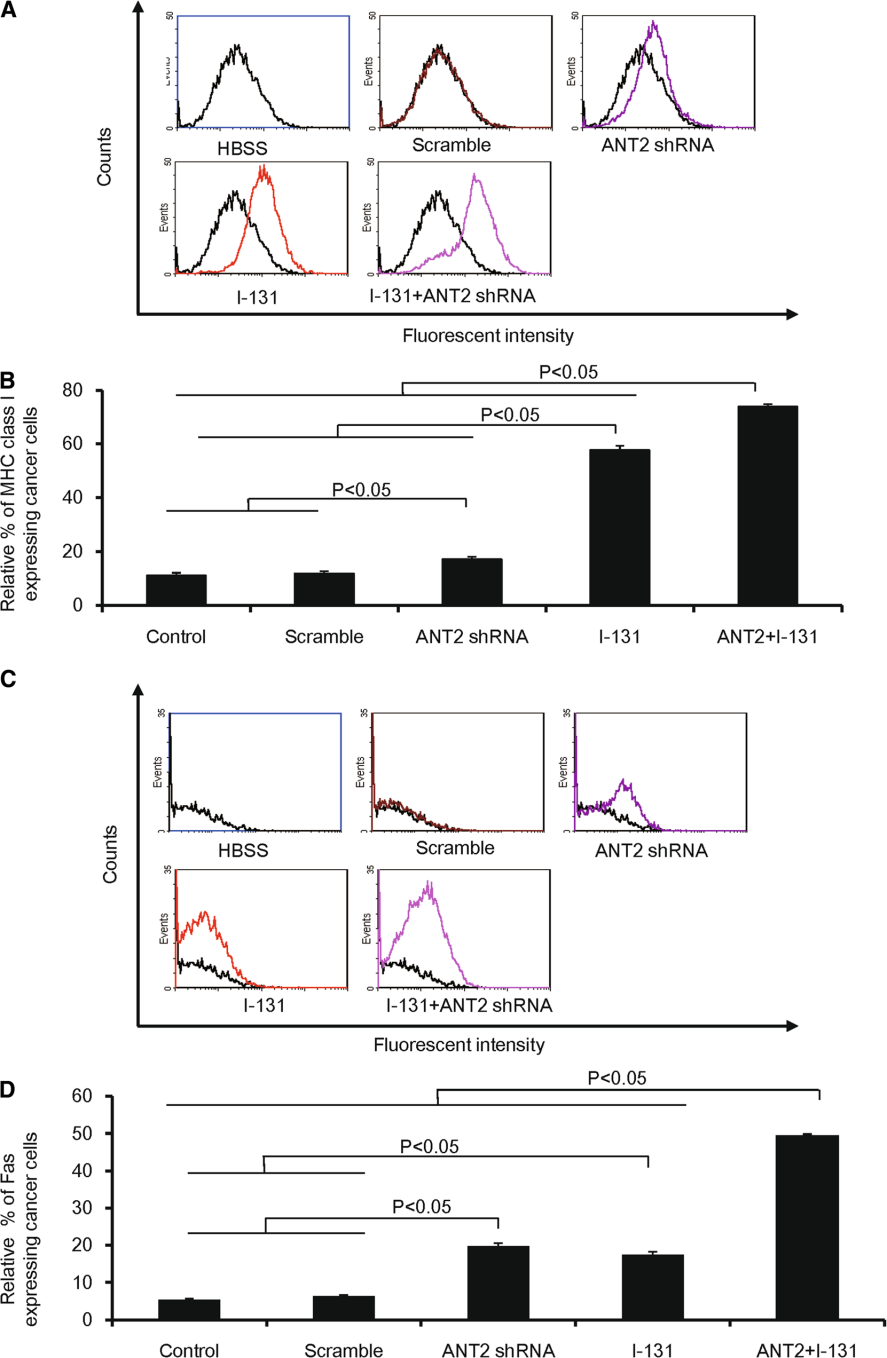
**The modulation of phenotypic markers in CT26/NF cells treated with ANT shRNA and hNIS radioiodine combination therapy. (A)** and **(C)** Representative flow cytometry data for MHC class I and Fas are shown. **(B)** and **(D)** The Y axis indicates the relative increase in MHC class I and Fas expression levels in cancer cells. A total of 10,000 cells were analyzed, and the relative% depicts the increased percentage of surface marker gene expression of treated cells compared with control. The data shown are the mean of triplicate experiments; the bars represent the mean ± SD.

Treatment with ANT2 shRNA or I-131 alone induced a 1.4- and 4.8-fold increase in MHC class I expression levels compared with control (Figures 3A and B; P<0.05, HBSS or scramble versus ANT2 shRNA; P<0.05, HBSS, scramble or ANT2 shRNA versus I-131). MHC class I expression levels were strongly increased after combination treatment relative to single treatment (ANT2 shRNA, I-131, and the combination induced expression levels of 17.2±1.0%, 57.8±1.7%, and 73.9±1.0%, respectively; p<0.05, ANT2 shRNA or I-131 versus ANT2 shRNA+I-131).

With regard to Fas expression, single treatment with ANT2 shRNA or I-131 showed an increase in Fas expression levels compared with control (Figures 3C and D; ANT2 shRNA and I-131 induced Fas expression levels of 19.8±0.8% and 17.5±0.5%, respectively; P<0.05 HBSS or scramble versus ANT2 shRNA; P<0.05 HBSS or scramble versus I-131). The combination treatment demonstrated a 2.4 (compared with ANT2 shRNA; P<0.05, ANT2 shRNA versus ANT2 shRNA+I-131) and a 3.1 (compared with I-131; P<0.05, I-131 versus ANT2 shRNA+I-131) -fold increase in Fas expression compared with single treatment.

**Figure 4:**
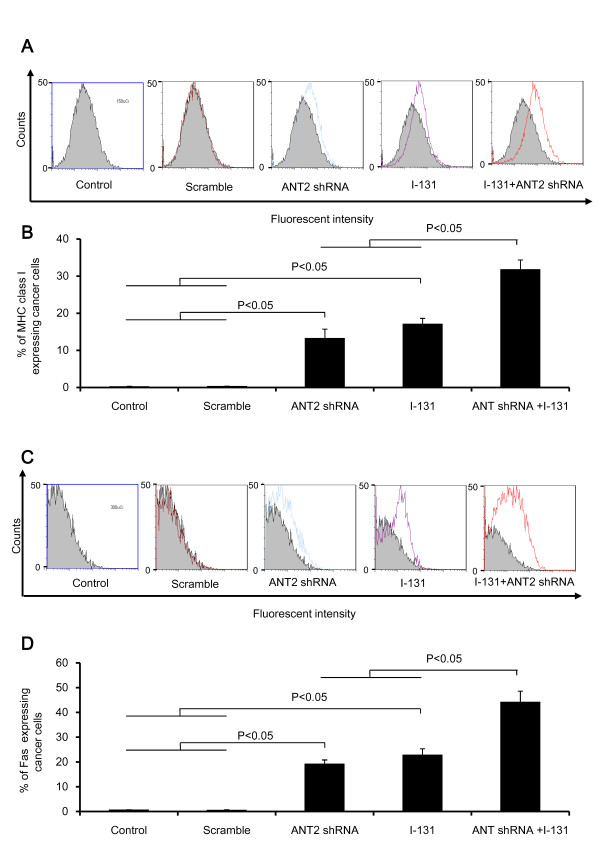
**The change in phenotypic markers in the CT26/NF tumor model treated with ANT shRNA and hNIS radioiodine combination therapy. (A)** and **(C)** Representative flow cytometry data for MHC class I and Fas are shown. **(B)** and **(D)** The Y axis indicates the relative increase of MHC class I and Fas expression in cancer cells. A total of 10,000 cells were analyzed, and the relative% depicts the increased percentage of surface marker gene expression of treated cells compared with control. The data shown are the mean of triplicate experiments; the bars represent the mean ± SD.

After the in vitro analyses, we tested whether the combination therapy could modulate the phenotypic markers of cells in the xenograft model. CT26/NF tumor-bearing mice were treated with scramble, ANT2 shRNA, I-131, and the combination through intratumoral and intravenous injection. As illustrated in Figures 4A and B, the percentage of MHC class I-expressing cells increased further after single treatment than in the control cells (scramble, ANT2 shRNA, and I-131 induced expression levels of 0.2±0.1%, 13.2±2.5%, and 17.1±1.5%, respectively; P<0.05, control or scramble versus ANT2 shRNA or I-131; P<0.05, ANT2 shRNA or I-131 versus combination). In addition, the combination therapy induced a significantly higher percentage of MHC class I expression levels than ANT2 shRNA or I-131 alone.

As shown in Figures 4C and D, the tumor cells treated with the combination therapy showed significantly higher expression levels of Fas than tumor cells treated with ANT2 shRNA or I-131 (19.2±1.6%, 22.7±2.5% and 44.1±4.4%, ANT2 shRNA, I-131, combination, respectively; P<0.05, control or scramble versus ANT2 shRNA or I-131, P<0.05, ANT2 shRNA or I-131 versus combination).

### Combined treatment induced remarkable antitumor effects in CT26/NF tumor-bearing mice

**Figure 5:**
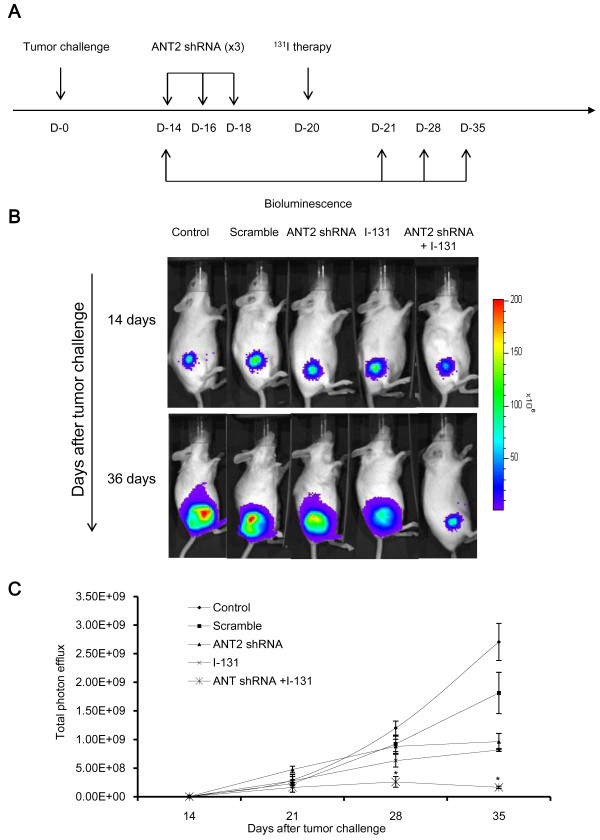
**In vivo visualization of the antitumor effects of ANT2 shRNA and hNIS radioiodine combination therapy. (A)** The in vivo tumor treatment schedule is shown. **(B)** The tumor growth inhibition effects were monitored in vivo using bioluminescent imaging. (**C**) The tumor growth quantification is shown. CT26/NF cells were transplanted s.c. into the right thighs of immunocompetent Balb/c mice. Fourteen days later, tumor growth was measured using bioluminescence. Then, the tumor-bearing mice were treated with scramble (supplemented with Lipofectamine 2000), ANT2 shRNA (supplemented with Lipofectamine 2000), I-131, and combination therapy according to a designated schedule through an intravenous or intratumoral route. The data shown are the mean of triplicate experiments; the bars represent the mean±SD (n = 7 mice/group).

To evaluate the therapeutic outcome after combination therapy, we performed the following procedure, which is depicted in Figure 5A. As illustrated in Figures 5B, C and Additional file 2: Figure S2, the tumor grew progressively in the control and scramble groups. However, single therapy resulted in a slight retardation of tumor growth; there was no difference in tumor growth inhibition between ANT2 shRNA and I-131. Interestingly, bioluminescence and tumor measurements showed remarkable tumor growth inhibition after combination therapy, and strong antitumor effects were sustained for 35 days (ANT2 shRNA or I-131 versus combination; P<0.05).

### Combination ANT2 shRNA and I-131 therapy induced a higher CTL killing activity against CT26/NF cells than single therapy

**Figure 6:**
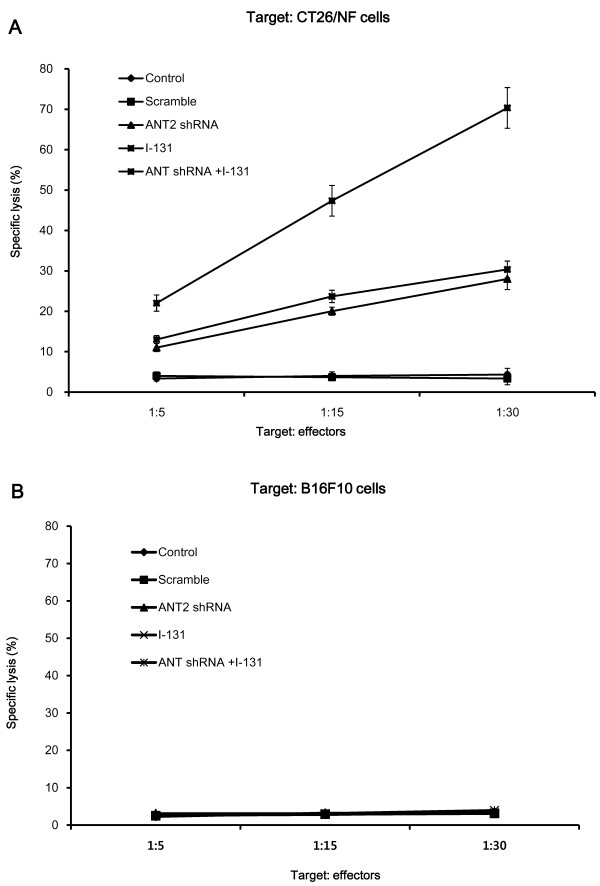
**The enhanced cytotoxic effects of CTLs on CT26/NF cancer cells by combination therapy.** Specific lysis was observed in **(A)** CT26/NF and **(B)** B16F10 cells. The splenocytes of treated mice were prepared and stimulated with IL-2 and irradiated CT26/NF and B16F10 cells for 3 days. Irradiated CT26/NF and B16F10 cells (target cells) were then incubated with splenocytes (effectors) at a T:E ratio of 1:5, 1:15, and 1:30 for 4 h in 96-well plates. The experiments were performed in triplicate, and the bars represent the mean ±SD; *, P<0.01; n = 7 mice/group.

The CTLs from mice receiving single therapy had enhanced killing activity compared with the control mice, and specific lysis (%) against CT26/NF cells was increased according to the different ratio of target/effectors (Figure 6A). The killing activity of the CTLs after combination therapy was most effective among the five experimental groups, showing 22±2.0%, 47±3.8%, and 70±5.0% specific lysis at target/effectors ratios of 1/5, 1/15, and 1/30, respectively. However, there was no specific lysis of CTLs against B16F10 cells in all treatment groups at target/effector ratios of 1/5, 1/15 and 1/30 (Figure 6B).

## Discussion

Efficient expression of the MHC I class molecule has proven to be a critical factor in the presentation of tumor antigen to CTLs [15,16]. Because the MHC class I gene expression levels are down-regulated in several cancers, members of this class can effectively escape immunosurveillance [17]. Similar to the MHC class I molecule, Fas (known as CD178 or CD95L) is a key molecule for apoptosis induction of cancer cells through interaction with the Fas ligand secreted by CTLs and the Fas receptor (CD95) on tumor cells [18].

Several reports have shown that external beam radiotherapy can concurrently up-regulate MHC class I and Fas gene expression levels in human or mouse cancer models in vitro and in vivo [19-22]. For example, Chakraborty et al. found that irradiated cancer cells highly express Fas receptor and ICAM-1 in a dose-dependent manner, and phenotypic modification augmented the susceptibility of cancer cells to CTLs [19]. Furthermore, Garnett et al. revealed that radiation with 10 Gy up-regulates Fas, ICAM-1, MUC-1, CEA, and MHC class I in human cancers [20]. In parallel with external radiation therapy, it was reported by our group that hNIS radioiodine gene therapy modulates the expression of surface markers and subsequently enhances anti-tumor immunity through an increased susceptibility of cancer cells to cytotoxic T cell (CTLs) [14].

Gene silencing through siRNA has proven to effectively knock down the expression of genes of interest in a wide range of cell types [23,24]. Recently, many groups have shown that siRNA-mediated cancer treatment has potential as a cancer therapy in vitro and in vivo [25-28]. Some reports have demonstrated the induction of the immune response (such as phenotype modification of cancer cells and an increased susceptibility of cancer cells to effector cells) by siRNA-mediated therapy. However, many researchers have successfully demonstrated the inhibition of specific genes involved in cancer progression with the siRNA technique. This study, focusing on the effect of the immune response induced by siRNA-mediated therapy in the tumor microenvironment, increases the limited data on the therapeutic outcome of conventional single therapy. Recently, in light of these concerns, we have shown that silencing ANT2 gene expression with siRNA effectively induces apoptosis in cancer cells and retards cancer progression [29]. More interestingly, the in vivo administration of ANT shRNA modulates the surface markers of cancer cells such as the MHC class I, Fas and ICAM-1; thus, it is an important molecule for inducing strong anti-tumor immunity [8].

In this study, we have successfully demonstrated that combination therapy with ANT2 shRNA and hNIS radioiodine gene results in a higher induction of apoptosis than single therapy in mouse colon cancer cells (Figure 3). In addition, this combination treatment led to the largest increase in the gene expression levels of MHC class I and Fas in vitro and in vivo (Figure 4). We speculate that the results described above occurred through the following mechanisms. hNIS radioiodine gene therapy induces cell death through cellular apoptosis or necrosis by the strong radiation of β-rays from I-131. Single treatment with I-131 increase the gene expression levels of MHC class I and Fas. Similar to hNIS radioiodine gene therapy, Jang et al. demonstrated that ANT2 shRNA therapy induces apoptotic cell death (through the potential disruption of mitochondrial membrane) and bystander effects. In the current study, we confirmed that ANT2 shRNA not only induced apoptotic cell death but also increased MHC class I and Fas gene expression levels. From these results, we hypothesize that the combination of ANT2 shRNA and I-131 lead to increased apoptotic cell death and MHC class I and Fas gene expression levels compared with single treatment through the additive effects of the respective single therapy agents in vitro and in vivo. In the case of in vivo therapy, bioluminescence showed that the combined treatment generated more marked therapeutic outcomes, while single therapy resulted in a slight tumor inhibition (Figure 5). Consistent with the in vivo antitumor effects of combination therapy, it was shown that CT26/NF cells from mice receiving combined treatment are most susceptible to CTLs (Figure 6).

Although we could not evaluate the full spectrum of antitumor immunity generated by combination therapy, we were able to speculate on the possible mechanism involved in the successful inhibition of tumor growth as well as in the strong generation of antitumor immunity of CTLs: 1) the combination of ANT2 shRNA and hNIS radioiodine gene therapy modulated phenotypic markers and strongly induced cell death through apoptosis or necrosis; 2) abundant peptides from dying cancer cells may have acted as antigenic peptides for professional antigen-presenting cells (APCs); 3) mature APCs (against cancer-derived peptides) may effectively stimulate tumor-specific effector cells, and these effectors will then recognize cancer cells with higher MHC class I and Fas expression levels. Finally, concurrent induction of both strong cell death and increased anti-tumor immunity by combination therapy may provide strong antitumor effects in living organisms. However, to extend this study, systemic delivery of hNIS and ANT shRNA should be investigated using a tissue-specific promoter and a viral system in an animal tumor model to further mimic the clinical situation.

## Conclusions

In conclusion, this is the first preclinical study to demonstrate that combined ANT2 shRNA and hNIS radioiodine gene therapy 1) induces strong apoptosis and markedly up-regulates MHC class I and Fas gene expression levels in cancer cells in vitro and in vivo and 2) increases most killing activity of CTLs and generates remarkable tumor growth inhibition in an immunocompromised mouse colon cancer model. These results provide alternative therapeutic strategies in cancer patients who are not highly responsive to conventional therapy.

## Abbreviations

CT26/NF: CT26/NIS-Fluc; ANT2: Adenine nucleotide translocator-2; hNIS: Human sodium iodide symporter; LDH: Lactate dehydrogenase; CTLs: Cytotoxic T cells; Fluc: Firefly luciferase; shRNA: Short hairpin RNA.

## Competing interests

The authors declare that they have no competing interests.

## Authors’ contributions

YC contributed to study conception and design, data analysis and interpretation and drafting the manuscript. HWL contributed to data acquisition, analysis, and interpretation. JL was involved in drafting and revising the manuscript for important intellectual content. YHJ contributed to study conception and design, data analysis and interpretation and drafting the manuscript. All authors read and approved the final manuscript.

## Pre-publication history

The pre-publication history for this paper can be accessed here:

http://www.biomedcentral.com/1471-2407/13/143/prepub

## Supplementary Material

Additional file 1: Figure S1 In vitro caspase-3 activity after treatment with ANT2 shRNA, I-131 and combination treatment. Detailed experimental procedures are described in the methods section. At 2 days after treatments, cells were lysed and an equal volume of a proluminescent substrate (DEVD), and cytosolic proteins were added to a white-walled 96-well plate and incubated at room temperature for 1 hour. The luminescence of each sample run in triplicate was measured in a plate-reading luminometer. The columns indicate the mean of triplicate experiments; the bars indicate the SD. Click here for file

Additional file 2: Figure S2 Tumor volume and weight measurements. (A) The tumor volume was defined as *V* = 1/2(*L* × *W*^2^), where L is length (longest dimension) and W is width (shortest dimension). (B) The tumor mass was extracted and weighed 35 days post-tumor challenge. The data shown are the mean of triplicate experiments; the bars represent the mean ± SD; n = 7 mice/group. Click here for file

## References

[B1] Gurunathan S Klinman DM Seder RA DNA vaccines: immunology, application, and optimization* Annu Rev Immunol 2000 18 927 974 10.1146/annurev.immunol.18.1.927 10837079

[B2] Rosenberg SA Yang JC Sherry RM Hwu P Topalian SL Schwartzentruber DJ Restifo NP Haworth LR Seipp CA Freezer LJ Inability to immunize patients with metastatic melanoma using plasmid DNA encoding the gp100 melanoma-melanocyte antigen Hum Gene Ther 2003 14 8 709 714 10.1089/104303403765255110 12804135 PMC2078240

[B3] Ferrone S Marincola FM Loss of HLA class I antigens by melanoma cells: molecular mechanisms, functional significance and clinical relevance Immunol Today 1995 16 10 487 494 10.1016/0167-5699(95)80033-6 7576053

[B4] Smyth MJ Godfrey DI Trapani JA A fresh look at tumor immunosurveillance and immunotherapy Nat Immunol 2001 2 4 293 299 10.1038/86297 11276199

[B5] Dudley ME Rosenberg SA Adoptive-cell-transfer therapy for the treatment of patients with cancer Nat Rev Cancer 2003 3 9 666 675 10.1038/nrc1167 12951585 PMC2305722

[B6] Dolce V Scarcia P Iacopetta D Palmieri F A fourth ADP/ATP carrier isoform in man: identification, bacterial expression, functional characterization and tissue distribution FEBS Lett 2005 579 3 633 637 10.1016/j.febslet.2004.12.034 15670820

[B7] Lunardi J Attardi G Differential regulation of expression of the multiple ADP/ATP translocase genes in human cells J Biol Chem 1991 266 25 16534 16540 1885585

[B8] Choi Y Jeon YH Jang JY Chung JK Kim CW Treatment with mANT2 shRNA enhances antitumor therapeutic effects induced by MUC1 DNA vaccination Mol Ther 2011 19 5 979 989 10.1038/mt.2010.235 21063392 PMC3098626

[B9] Chung JK Sodium iodide symporter: its role in nuclear medicine J Nucl Med 2002 43 9 1188 1200 12215558

[B10] De La Vieja A Dohan O Levy O Carrasco N Molecular analysis of the sodium/iodide symporter: impact on thyroid and extrathyroid pathophysiology Physiol Rev 2000 80 3 1083 1105 10893432 10.1152/physrev.2000.80.3.1083

[B11] Cho JY A transporter gene (sodium iodide symporter) for dual purposes in gene therapy: imaging and therapy Curr Gene Ther 2002 2 4 393 402 10.2174/1566523023347599 12477251

[B12] Chen L Altmann A Mier W Eskerski H Leotta K Guo L Zhu R Haberkorn U Radioiodine therapy of hepatoma using targeted transfer of the human sodium/iodide symporter gene J Nucl Med 2006 47 5 854 862 16644756

[B13] Jeon YH Choi Y Yoon SO Kim CW Chung JK Synergistic tumoricidal effect of combined hMUC1 vaccination and hNIS radioiodine gene therapy Mol Cancer Ther 2008 7 7 2252 2260 10.1158/1535-7163.MCT-08-0277 18645034

[B14] Jeon YH Choi Y Kim HJ Kim CW Jeong JM Lee DS Chung JK Human sodium iodide symporter gene adjunctive radiotherapy to enhance the preventive effect of hMUC1 DNA vaccine Int J Cancer 2007 121 7 1593 1599 10.1002/ijc.22837 17565743

[B15] Gilboa E How tumors escape immune destruction and what we can do about it Cancer Immunol Immunother 1999 48 7 382 385 10.1007/s002620050590 10501851 PMC11037178

[B16] Garcia-Lora A Algarra I Garrido F MHC class I antigens, immune surveillance, and tumor immune escape J Cell Physiol 2003 195 3 346 355 10.1002/jcp.10290 12704644

[B17] Bubenik J Tumour MHC class I downregulation and immunotherapy (Review) Oncol Rep 2003 10 6 2005 2008 14534734

[B18] Kojima H Shinohara N Hanaoka S Someya-Shirota Y Takagaki Y Ohno H Saito T Katayama T Yagita H Okumura K Two distinct pathways of specific killing revealed by perforin mutant cytotoxic T lymphocytes Immunity 1994 1 5 357 364 10.1016/1074-7613(94)90066-3 7533644

[B19] Chakraborty M Abrams SI Camphausen K Liu K Scott T Coleman CN Hodge JW Irradiation of tumor cells up-regulates Fas and enhances CTL lytic activity and CTL adoptive immunotherapy J Immunol 2003 170 12 6338 6347 12794167 10.4049/jimmunol.170.12.6338

[B20] Garnett CT Palena C Chakraborty M Tsang KY Schlom J Hodge JW Sublethal irradiation of human tumor cells modulates phenotype resulting in enhanced killing by cytotoxic T lymphocytes Cancer Res 2004 64 21 7985 7994 10.1158/0008-5472.CAN-04-1525 15520206

[B21] Demaria S Bhardwaj N McBride WH Formenti SC Combining radiotherapy and immunotherapy: a revived partnership Int J Radiat Oncol Biol Phys 2005 63 3 655 666 10.1016/j.ijrobp.2005.06.032 16199306 PMC1489884

[B22] Gulley JL Arlen PM Bastian A Morin S Marte J Beetham P Tsang KY Yokokawa J Hodge JW Menard C Combining a recombinant cancer vaccine with standard definitive radiotherapy in patients with localized prostate cancer Clin Cancer Res 2005 11 9 3353 3362 10.1158/1078-0432.CCR-04-2062 15867235

[B23] Fire A Xu S Montgomery MK Kostas SA Driver SE Mello CC Potent and specific genetic interference by double-stranded RNA in Caenorhabditis elegans Nature 1998 391 6669 806 811 10.1038/35888 9486653

[B24] Hannon GJ RNA interference Nature 2002 418 6894 244 251 10.1038/418244a 12110901

[B25] Wu H Hait WN Yang JM Small interfering RNA-induced suppression of MDR1 (P-glycoprotein) restores sensitivity to multidrug-resistant cancer cells Cancer Res 2003 63 7 1515 1519 12670898

[B26] Urban-Klein B Werth S Abuharbeid S Czubayko F Aigner A RNAi-mediated gene-targeting through systemic application of polyethylenimine (PEI)-complexed siRNA in vivo Gene Ther 2005 12 5 461 466 10.1038/sj.gt.3302425 15616603

[B27] Crnkovic-Mertens I Hoppe-Seyler F Butz K Induction of apoptosis in tumor cells by siRNA-mediated silencing of the livin/ML-IAP/KIAP gene Oncogene 2003 22 51 8330 8336 10.1038/sj.onc.1206973 14614456

[B28] Goldberg MS Xing D Ren Y Orsulic S Bhatia SN Sharp PA Nanoparticle-mediated delivery of siRNA targeting Parp1 extends survival of mice bearing tumors derived from Brca1-deficient ovarian cancer cells Proc Natl Acad Sci U S A 2011 108 2 745 750 10.1073/pnas.1016538108 21187397 PMC3021044

[B29] Jang JY Choi Y Jeon YK Kim CW Suppression of adenine nucleotide translocase-2 by vector-based siRNA in human breast cancer cells induces apoptosis and inhibits tumor growth in vitro and in vivo Breast Cancer Res 2008 10 1 R11 10.1186/bcr1857 18267033 PMC2374967

