# Supplementary figures and images for: The combination of ANT2 shRNA and hNIS radioiodine gene therapy increases CTL cytotoxic activity through the phenotypic modulation of cancer cells: combination treatment with ANT2 shRNA and I-131
Source: BMC Cancer. 2013 Mar 22;13:143. doi: 10.1186/1471-2407-13-143 (PMC3653759; doi:10.1186/1471-2407-13-143)

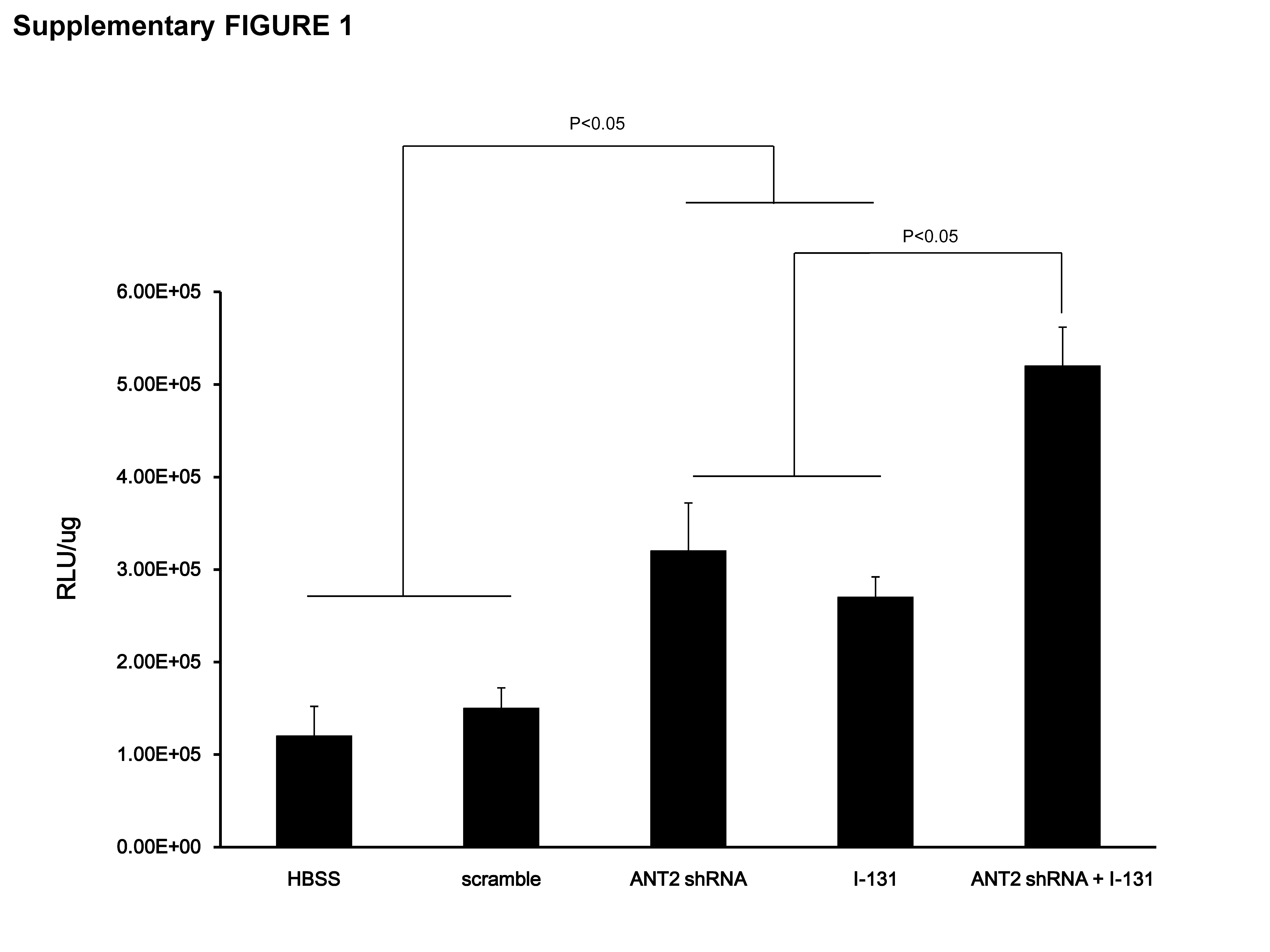

Supplement: Additional file 1: Figure S1 — In vitro caspase-3 activity after treatment with ANT2 shRNA, I-131 and combination treatment. Detailed experimental procedures are described in the methods section. At 2 days after treatments, cells were lysed and an equal volume of a proluminescent substrate (DEVD), and cytosolic proteins were added to a white-walled 96-well plate and incubated at room temperature for 1 hour. The luminescence of each sample run in triplicate was measured in a plate-reading luminometer. The columns indicate the mean of triplicate experiments; the bars indicate the SD. [file 1471-2407-13-143-S1.tiff]

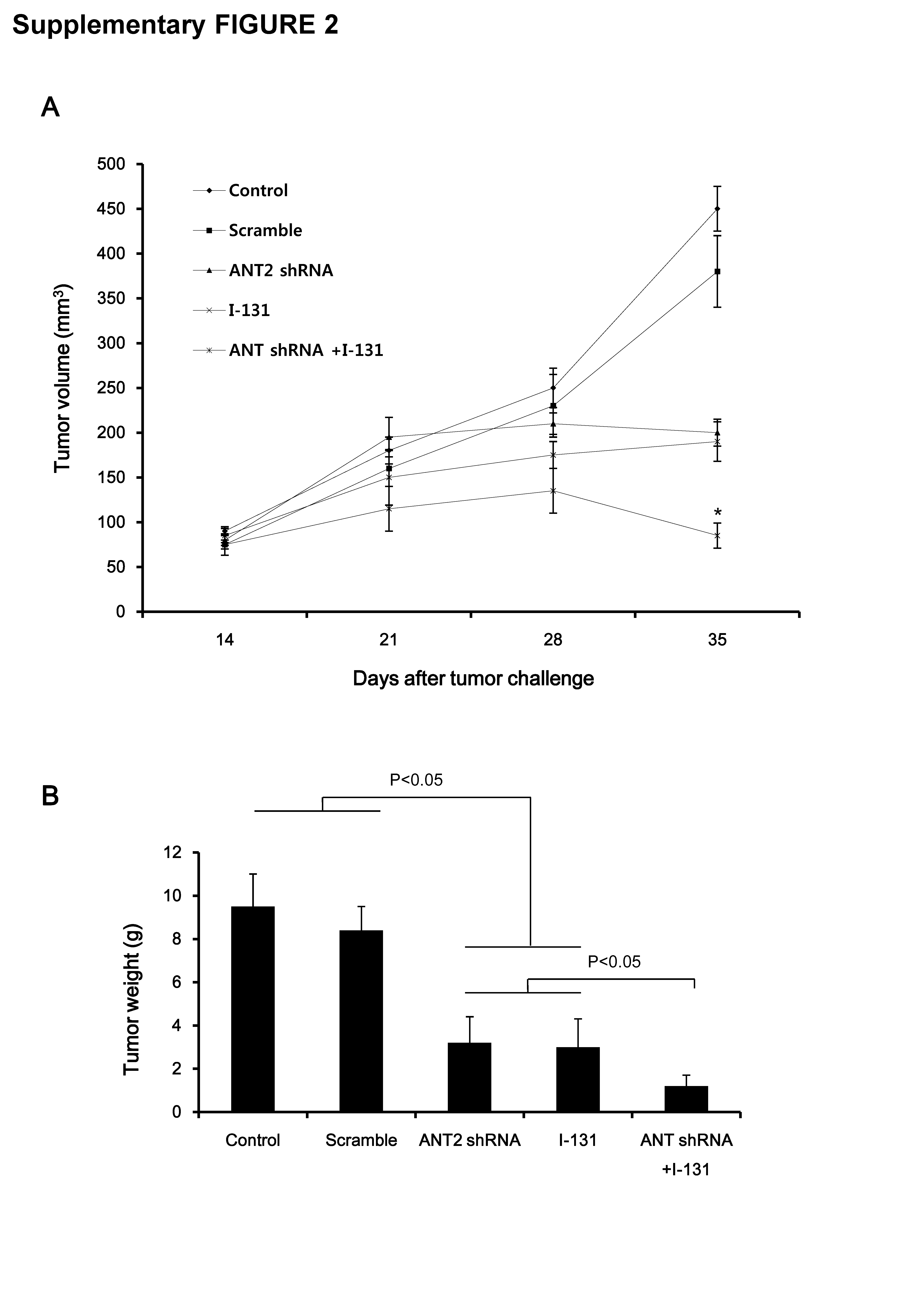

Supplement: Additional file 2: Figure S2 — Tumor volume and weight measurements. (A) The tumor volume was defined as V = 1/2(L × W2), where L is length (longest dimension) and W is width (shortest dimension). (B) The tumor mass was extracted and weighed 35 days post-tumor challenge. The data shown are the mean of triplicate experiments; the bars represent the mean ± SD; n = 7 mice/group. [file 1471-2407-13-143-S2.tiff]
